# Investigation of Endotracheal Tube Versus Laryngeal Mask Airway Emergence Times for Surgical Patients

**DOI:** 10.7759/cureus.85340

**Published:** 2025-06-04

**Authors:** Samir M Patel, Rayyan Bhutta, Michelle Pershing, John Dillis, John O Elliott, Matthias J Franzen

**Affiliations:** 1 Anesthesiology and Perioperative Medicine, OhioHealth Doctors Hospital, Columbus, USA; 2 Anesthesiology and Perioperative Medicine, Ohio University Heritage College of Osteopathic Medicine, Dublin, USA; 3 Research and Development, OhioHealth Research Institute, Columbus, USA; 4 Biostatistics, OhioHealth Research Institute, Columbus, USA; 5 Research, OhioHealth Research Institute, Columbus, USA

**Keywords:** academic anesthesiology, emergence time, ett, general anesthesia, general endotracheal anesthesia, laryngeal mask airway (lma), placement of endotracheal tube (ett)

## Abstract

Introduction: During surgical procedures requiring general anesthesia, an airway device is often placed to allow for adequate ventilation of the patient. Most commonly, endotracheal tubes (ETTs) or laryngeal mask airways (LMAs) are placed by the anesthesia provider. The difference between these two methods of ventilation is based on the location; ETTs pass through the glottis into the trachea, whereas the LMA sits above the glottis. This difference in placement may lend benefit to LMAs over ETTs, one of which includes a faster removal of the airway device. That is, after the surgical procedure is completed, there is a period when the anesthetic agent is turned off in preparation for the patient's awakening and subsequent removal of the airway device, also known as emergence. In this study, we aim to elucidate whether a difference in emergence times exists between using an ETT or LMA after general anesthesia.

Methods: This retrospective study was conducted at OhioHealth Doctors Hospital, located in Columbus, Ohio, USA. Patients 18 years or older who underwent index surgery under general anesthesia between July 1, 2019 and December 31, 2019 were included. Patients were categorized based on the airway type (ETT or LMA). Clinical data and surgical outcomes were analyzed using descriptive statistics and appropriate statistical tests.

Results: A total of 629 patients (282 ETT, 347 LMA) were included. The mean emergence time in the LMA group was found to be significantly shorter compared to that in the ETT group (5.6 vs 7.2 minutes, p<0.001). The LMA also correlated with reduced anesthesia duration, operating room (OR) time, and procedure time, with all p-values <0.001. Regression models identified the airway type and other various independent variables as predictors of emergence time and OR duration. The adjusted R² for emergence time was 5.90%, whereas the adjusted R² for OR duration was 41.26%.

Conclusion: LMA use was associated with significantly shorter emergence times and operative times compared to ETTs. These findings suggest potential clinical benefits for patients, as well as workflow advantages with LMA use in appropriate surgical settings. Further prospective research is warranted to evaluate clinical outcomes, as well as cost-effectiveness.

## Introduction

The administration of general anesthesia during surgical procedures necessitates careful consideration of airway management to ensure optimal patient outcomes. Among the various methods employed by anesthesia providers, the choice between endotracheal tubes (ETTs) and laryngeal mask airways (LMAs) stands as a crucial decision. The fundamental distinction lies in the placement of these airway devices; while ETTs traverse the glottic opening and extend into the trachea, LMAs rest above the glottis. This disparity in anatomical positioning has prompted exploration into potential advantages of one method over the other.

During surgical procedures requiring general anesthesia, an airway device is often placed to allow for adequate ventilation of the patient. Most commonly, ETTs or LMAs are placed by the anesthesia provider. Depending on the type of procedure or patient factors, intubation may be indicated. Such indications of intubation include, but are not limited to, patients who are at risk of aspiration, procedures involving body cavities (intrathoracic and intra-abdominal) [1], cardiac or respiratory arrest, failure to protect the airway from aspiration, inadequate oxygenation or ventilation, and impending or existing airway obstruction [2]. However, LMAs have been demonstrated as a means of safely securing the airway for general anesthesia required during laparoscopic surgery [3] or implantation of positive pressure ventilation [4].

Prior to surgery, a patient is given anesthetic agents to induce unconsciousness. When the patient is in a deep enough anesthetic state, the airway device is then inserted. If an ETT is utilized, neuromuscular blocking agents are routinely given to paralyze the vocal cords, optimizing conditions for intubation. If an LMA is used, no neuromuscular blocking agents are used for airway placement. After the airway is secured, typically a volatile anesthetic is started to maintain the depth of anesthesia and to allow for the surgery to proceed. After the surgical procedure is completed, there is a period when the anesthetic agent is turned off in preparation for the patient's awakening and subsequent removal of the airway device, known as emergence. Emergence time is defined as the passive process of the return to consciousness after discontinuing administration of anesthetic and adjuvant agents at the end of the surgical procedure. Literature exists comparing the emergence times between the use of ETTs and that of LMAs; however, it is ambiguous. One small study of 72 patients demonstrated that there was no difference in emergence times between the two when total intravenous anesthesia (TIVA) was used to maintain general anesthesia for trauma-related orthopedic surgery [5]. Another study of 381 patients undergoing elective peripheral surgery also found no difference between the duration of the end of surgery to removal of the airway device; however, in this case they utilized desflurane or isoflurane in conjunction with nitrous oxide to maintain general anesthesia [6]. In contrast, Todd demonstrated in a retrospective review of 157 patients that LMAs can show benefit in general anesthesia for dentoalveolar surgery by having a shorter procedure time and faster recovery time, when compared to ETTs [7]. Although the Todd study demonstrated the benefit of LMAs, this finding was limited to dentoalveolar surgery, which is a specific and relatively less invasive procedure. The contradictory results of the studies may be explained by the different anesthetic techniques used (TIVA vs inhaled agents), surgical procedure types, or patient populations, suggesting that the results in these studies may not be generalized. Consequently, there remains a lack of clarity as to whether the choice of airway device independently influences emergence time, which this study aims to address. 

The literature comparing ETTs to LMAs to date consists of relatively small sample sizes in limited/specific types of surgical interventions. The high volume of surgeries across specialties at Doctors Hospital allows a more robust comparison of ETTs and LMAs across surgical specialties. The purpose of this retrospective study is to compare emergence times between two groups of surgical patients who underwent general anesthesia with sevoflurane: those who had an ETT placed and those who were managed using an LMA. If a difference does indeed exist, considerations can be made as to how to utilize this information to potentially decrease operating room (OR) time and cost.

One specific area of interest in this regard is the emergence time, the duration from the completion of the surgical procedure to the removal of the airway device. The hypothesis guiding this investigation posits that LMA usage may be associated with faster emergence times compared to ETTs. The pivotal moments post-surgery involve the cessation of anesthetic agents in anticipation of the patient's awakening and the subsequent removal of the airway device. This study, therefore, seeks to comprehensively evaluate the emergence times based on the method of airway management-comparing ETT and LMA groups, both overall and specifically for those administered nitrous oxide.

The research is structured around three principal aims. The first aim compares emergence times between ETT and LMA groups, investigating potential variations and associations with patient and provider characteristics using regression methods. The second aim extends the inquiry to explore broader operative data, encompassing anesthesia duration, procedure time, and overall OR time in both ETT and LMA groups. The third aim seeks to establish a comprehensive understanding of the relationships between emergence times and various operative outcomes, hypothesizing that expedited emergence may translate to reduced anesthesia duration, procedure times, and OR times.

In the following sections, we delve into the methods, results, and discussions that illuminate the nuanced interplay between airway management methods and their impact on the critical phases of surgical procedures under general anesthesia. Through this investigation, we aspire to contribute valuable insights that may inform clinical practices and enhance patient care in the realm of anesthetic management.

## Materials and methods

This retrospective study aims to investigate the impact of ETTs versus LMAs on various anesthesia and surgical outcomes in surgical patients aged 18 years and older. The study was conducted at OhioHealth Doctors Hospital, Department of Anesthesiology, located in Columbus, Ohio, United States. The study subjects were identified through a comprehensive query of the OhioHealth database, specifically CareConnect, conducted by Quality and Patient Safety (QPS). The initial patient list generated was meticulously reviewed and validated by the investigator or designated study staff to ensure accuracy and adherence to inclusion/exclusion criteria.

The inclusion criteria encompass surgical patients aged 18 years or older, who underwent an index surgery between July 1, 2019, and December 31, 2019, at OhioHealth Doctors Hospital, with general anesthesia administered using sevoflurane. Exclusion criteria involve cases of emergent surgery, patients who remained intubated post-procedure, use of multiple airways, multiple volatile anesthetics, continuous IV anesthetics, charting abnormalities, and exclusion of any surgeries conducted after the index surgery.

The anticipated study sample comprises a maximum of 2,071 patients, evenly distributed between ETT and LMA groups. This estimation is based on a preliminary analysis of aggregate data. However, the final sample size was narrowed down to 629 patients (282 ETT and 347 LMA) due to the implementation of the inclusion criteria and the time frame of the study. In case of a higher-than-expected number meeting inclusion criteria, a protocol amendment will be submitted to adjust the sample size accordingly.

Data collected for analysis included demographics, diagnosis/diagnosis codes, discharge summaries, treatment procedures, treatment-related dates, billing information, drug/device usage details, treatment provider information, and surgical/procedure notes. Notably, the study involves the collection of Protected Health Information (PHI).

Patients were categorized into ETT or LMA groups based on documented airway management methods. Independent variables for analysis include patient demographics, medical history (including ASA category, smoking status, and history of sleep apnea), operative data (including adjunct anesthesia, anxiolytics, and analgesia usage), and surgical specialty. Endpoints for analysis, addressing specific study aims, include emergence time (minutes), anesthesia duration, procedure time, and total operative time. All endpoints were calculated from discrete time/date fields in Epic using the Research Electronic Data Capture (REDCap) platform, ensuring systematic and standardized data abstraction.

Statistical analysis was conducted using JASP and R statistical software (R Foundation for Statistical Computing, Vienna, Austria). Normality of variables such as anesthesia duration, total operative time, and emergence time was assessed using statistical tests such as the Shapiro-Wilk test. Based on the distribution of the data for emergence times, the comparisons made between the ETT and LMA groups were performed using the Mann-Whitney U test. Chi-square tests were used to analyze categorical variables, whereas linear regression was used to assess the relationships between the airway type and the outcomes. Variables that were not statistically significant at the p<0.05 value were removed from the models. These tests were used to provide correlation coefficients (R), confidence intervals and p-values, with a significance threshold of p<0.05 being used for all statistical comparisons. A formal power analysis was not conducted; however, the sample size was determined based on the total number of cases due to the inclusion criteria within the selected timeframe. The final sample size of 629 still represents a reasonably large data set for retrospective analysis, which allows for meaningful statistical comparisons. 

## Results

All study data were summarized with descriptive statistics. Continuous variables were summarized with means, standard deviations, medians, and ranges. Categorical variables were summarized with counts and percentages. Data was summarized overall and separately by ETT and LMA groups and overall and separately for those receiving nitrous oxide.

Emergence time was compared between groups using the Mann-Whitney test for non-normally distributed data. Multilinear regression methods were used to determine whether emergence times differed as a function of patient demographics or characteristics, operative data, or provider characteristics (Table 1). All variables in the univariate analyses with p-values ≤ 0.100 were included in the first model. Those variables not significant at the p-values ≤ 0.05 level were then removed for the reduced model.

**Table 1:** Demographic and Clinical Characteristics SD: Standard Deviation; BMI: Body Mass Index; n: number; ASA: American Society of Anesthesiologists; ETT: Endotracheal Tube; LMA: Laryngeal Mask Airway

Demographics	ETT (n=282)	LMA (n=347)	p-value
Age, mean ± SD	48.8 ± 16.2	49.9 ± 15.7	0.31
Race	-	-	0.73
Caucasian	216 (76.6)	276 (79.5)	-
African American	35 (12.4)	35 (10.1)	-
Asian	6 (2.1)	9 (2.6)	-
Declined or other	25 (8.9)	27 (7.8)	-
BMI, mean ± SD	31.3 ± 8.29	30.5 ± 7.30	0.38
Smoking Status	-	-	-
Current	79 (28.0)	123 (35.4)	0.10
Former	70 (24.8)	86 (24.8)	0.10
Never	133 (47.2)	138 (39.8)	0.10
Positive for apnea, n (%)	37 (13.1)	47 (13.5)	1.00
ASA Category, n (%)	-	-	-
ASA I	4 (1.4)	7 (2.0)	0.58
ASA II	155 (55.0)	172 (49.6)	0.58
ASA III	116 (41.1)	159 (45.8)	0.58
ASA IV	7 (2.5)	9 (2.6)	0.58
Pre-operative regional anesthetic, n (%)	63 (22.3)	27 (7.8)	<0.001
Intraoperative narcotics, n (%)	216 (76.6)	156 (45.0)	<0.001
Nitrous oxide, n (%)	59 (20.9)	95 (27.4)	0.08

Results with a p-value less than or equal to 0.05 are considered statistically significant. Statistical analyses were conducted using JASP (JASP Team, 2023, version 0.17.1) and R 4.3.0.

The emergence time (minutes) between ETT and LMA groups was compared, and regression methods were used to determine whether emergence time differs based on patient or provider characteristics. Emergence time was significantly (p<0.001) shorter in the LMA group than the ETT group, regardless of NO usage (Table 2).

**Table 2:** Emergence Time between the ETT and LMA Groups Statistical analysis was performed using the Mann-Whitney test SD: Standard Deviation; N: Number; NO: Nitrous Oxide; ETT: Endotracheal Tube; LMA: Laryngeal Mask Airway; Min: Minimum; Max: Maximum

Emergence Time	ETT	LMA	p-value
Emergence time, minutes	-	-	-
Mean	7.2	5.6	<0.001
SD	5.14	4.68	<0.001
Median	6.0	4.5	<0.001
Min, Max	0.10, 36.1	0.10, 30.0	<0.001
N	282	347	<0.001
Emergence time, minutes, excluding NO	-	-	-
Mean	6.9	5.6	<0.001
SD	4.9	4.7	<0.001
Median	5.9	4.5	<0.001
Min, Max	0.10, 36.1	0.10, 30.0	<0.001
N	223	252	<0.001
Emergence time, minutes, NO group alone	-	-	-
Mean	8.4	5.5	<0.001
SD	5.8	4.6	<0.001
Median	6.8	4.9	<0.001
Min, Max	0.60, 29.3	0.10, 27.0	<0.001
N	59	95	<0.001

For both the full and reduced models, the variables of tube type (LMA vs. ETT), OR time, narcotics, and urology were significant (p<0.001). The full model had a lower adjusted R2 than the reduced model (5.88% vs 5.90%) indicating that the additional input variables were not adding value to the model. In the reduced model, the variables of OR time and urology had positive regression coefficients indicating an increase in emergence time. Furthermore, the tube type had a negative regression coefficient, indicating a reduction in time (Tables 3, 4).

**Table 3:** Regression Emergence Time: Full Model Full Model Adjusted R2 = 5.88%, p < 0.001 B: Slope of the Regression Line; CI: Confidence Interval; ETT: Endotracheal Tube; LMA: Laryngeal Mask Airway; OR: Operating Room

Full Model	B	95% CI	p-value
Constant	5.50	3.66 – 7.34	< 0.001
Tube type (ETT vs LMA)	-1.69	-2.63 – -0.74	< 0.001
OR time	0.01	0.01 – 0.02	0.002
Smoking	0.04	-0.40 – 0.48	0.863
Regional	-0.88	-2.11 – 0.36	0.163
Narcotics	-1.26	-2.11 – -0.42	0.003
Nitrous oxide	0.62	-0.26 – 1.51	0.167
General Surgery	0.75	-0.53 – 2.03	0.250
Urology	2.17	0.84 – 3.50	0.001
Orthopedics	0.66	-0.70 – 2.01	0.344

**Table 4:** Regression Emergence Time: Reduced Model Reduced Model Adjusted R2 = 5.90%, p < 0.001 B: Slope of the Regression Line; CI: Confidence Interval; ETT: Endotracheal Tube; LMA: Laryngeal Mask Airway; OR: Operating Room

Reduced Model	B	95% CI	p-value
Constant	6.23	4.92 – 7.53	< 0.001
Tube type (ETT vs LMA)	-1.64	-2.53 – -0.74	< 0.001
OR time	0.01	0.00 – 0.02	0.003
Smoking	-	-	-
Regional	-	-	-
Narcotics	-1.12	-1.95 – -0.30	0.008
Nitrous oxide	-	-	-
General Surgery	-	-	-
Urology	1.68	0.76 – 2.60	< 0.001
Orthopedics	-	-	-

Operative data (anesthesia duration, procedure time, OR time) between ETT and LMA groups were compared. The operative data times were significantly (<0.001) reduced in the LMA group compared to the ETT group, regardless of the usage of NO during the operations (Tables 5-7). On average, the LMA reduced 57 minutes in anesthesia duration, 56 minutes in OR duration, and 49 minutes in procedure time. The consistent results that were seen regardless of NO usage indicate that the efficacy of LMAs is independent of supplementation via NO, and the LMA plays a key role in influencing operative efficiency.

**Table 5:** Summary of Operative Data SD: Standard Deviation; N: Number; ETT: Endotracheal Tube; LMA: Laryngeal Mask Airway; Min: Minimum; Max: Maximum

Operative Data	ETT	LMA	p-value
Anesthesia duration, minutes	-	-	-
Mean	138.71	81.44	<0.001
SD	59.42	38.53	<0.001
Median	131.00	68.00	<0.001
Min, Max	48.00, 382.00	36.00, 242.00	<0.001
N	282	347	<0.001
Operating room duration, minutes	-	-	-
Mean	133.11	76.61	<0.001
SD	58.70	37.96	<0.001
Median	124.50	63.00	<0.001
Min, Max	41.00, 376.00	33.00, 236.00	<0.001
N	282	347	<0.001
Procedure time, minutes	-	-	-
Mean	95.01	46.01	<0.001
SD	56.62	34.05	<0.001
Median	86.50	33.00	<0.001
Min, Max	16.00, 331.00	11.00, 192.00	<0.001
N	282	347	<0.001

**Table 6:** Summary of Operative Data (Excluding NO) SD: Standard Deviation; N: Number; NO: Nitrous Oxide; ETT: Endotracheal Tube; LMA: Laryngeal Mask Airway; Min: Minimum; Max: Maximum

Operative Data (NO Excluded)	ETT	LMA	p-value
Anesthesia duration, minutes	-	-	-
Mean	138.28	79.05	<0.001
SD	60.50	36.24	<0.001
Median	129.00	66.00	<0.001
Min, Max	48.00, 382.00	36.00, 242.00	<0.001
N	223	252	<0.001
Operating room duration, minutes	-	-	-
Mean	132.71	74.23	<0.001
SD	59.74	35.58	<0.001
Median	123.00	62.00	<0.001
Min, Max	41.00, 376.00	33.00, 236.00	<0.001
N	223	252	<0.001
Procedure time, minutes	-	-	-
Mean	96.00	43.97	<0.001
SD	58.27	31.18	<0.001
Median	85.00	33.00	<0.001
Min, Max	20.00, 331.00	11.00, 192.00	<0.001
N	223	252	<0.001

**Table 7:** Summary of Operative Data (NO Group Only) SD: Standard Deviation; N: Number; NO: Nitrous Oxide; ETT: Endotracheal Tube; LMA: Laryngeal Mask Airway; Min: Minimum; Max: Maximum

Operative Data (NO Only)	ETT	LMA	p-value
Anesthesia duration, minutes	-	-	-
Mean	140.32	87.78	<0.001
SD	55.63	43.60	<0.001
Median	136.00	73.00	<0.001
Min, Max	55.00, 273.00	41.00, 235.00	<0.001
N	59	95	<0.001
Operating room duration, minutes	-	-	-
Mean	134.61	82.91	<0.001
SD	55.05	43.24	<0.001
Median	131.00	70.00	<0.001
Min, Max	56.00, 268.00	34.00, 232.00	<0.001
N	59	95	<0.001
Procedure time, minutes	-	-	-
Mean	91.27	51.41	<0.001
SD	50.20	40.40	<0.001
Median	89.00	35.00	<0.001
Min, Max	16.00, 233.00	12.00, 185.00	<0.001
N	59	95	<0.001

A statistically significant difference (<0.001) was seen when comparing the distribution of surgical specialties between patients who received ETTs compared to LMAs. General Surgery made up the largest portion of surgical specialties that used ETTs at 53.5%, while Orthopedics and Urology were more commonly using LMAs, at 32.6% and 34.0% respectively. This trend likely reflects the correlation in airway selection in relation to procedural duration, and the requirements for patient positioning during procedures (Table 8) [8].

**Table 8:** Summary of Provider Characteristics a: Includes surgeries categorized as general surgery, general robotics, ophthalmology, and general vascular b: Includes surgeries categorized as orthopedics or podiatry c: Includes surgeries categorized as OB-GYN or GYN-robotics d: Includes surgeries categorized as urology or uro-robotics n: Number; ETT: Endotracheal Tube; LMA: Laryngeal Mask Airway

Surgery Type, n (%)	ETT (n=282)	LMA (n=347)	p-value
General Surgery^a^	151 (53.5)	74 (21.3)	<0.001
Orthopedics^b^	58 (20.6)	113 (32.6)	<0.001
OB-GYN^c^	34 (12.1)	42 (12.1)	<0.001
Urology^d^	39 (13.8)	118 (34.0)	<0.001

Given that we hypothesize that faster emergence times will result in reduced anesthesia duration, procedure times, and OR times, we determined the association between emergence times and operative outcomes (Tables 9, 10).

**Table 9:** Regression OR Time: Reduced Model Reduced Model Adjusted R2 = 41.26%, p < 0.001 B: Slope of the Regression Line; CI: Confidence Interval; ETT: Endotracheal Tube; LMA: Laryngeal Mask Airway

Reduced Model	B	95% CI	p-value
Constant	93.81	84.15 – 103.46	< 0.001
Tube type (ETT vs LMA)	-41.90	-49.49 – -34.32	< 0.001
Emergence time	1.03	0.34 – 1.72	0.004
Smoking	-	-	-
Regional	50.06	39.87 – 60.25	< 0.001
Narcotics	23.18	15.89 – 30.48	< 0.001
Nitrous oxide	-	-	-
General Surgery	-	-	-
Urology	-	-	-
Orthopedics	14.21	6.27 – 22.16	< 0.001

**Table 10:** Regression OR Time: Full Model Full Model Adjusted R2 = 41.47%, p < 0.001 B: Slope of The Regression Line; CI: Confidence Interval; ETT: Endotracheal Tube; LMA: Laryngeal Mask Airway

Full Model	B	95% CI	p-value
Constant	88.20	72.86 – 103.54	< 0.001
Tube type (ETT vs LMA)	-39.35	-47.23 – -31.46	< 0.001
Emergence time	1.10	0.40 – 1.80	0.002
Smoking	2.40	-1.51 – 6.31	0.229
Regional	48.59	38.30 – 58.88	< 0.001
Narcotics	22.27	14.91 – 29.62	< 0.001
Nitrous oxide	0.70	-7.21 – 8.60	0.862
General Surgery	3.46	-7.92 – 14.84	0.551
Urology	-7.03	-18.97 – 4.91	0.248
Orthopedics	13.37	1.31 – 25.43	0.030

For both the full and reduced models, the variables of tube type, regional, and narcotics were significant (p<0.001). The reduced model had a lower adjusted R2 than the full model (41.3% vs 41.5%) indicating that the additional input variables are adding value to the model. In the reduced model, the variables of emergence time, regional, and narcotics had positive regression coefficients indicating they add to OR time. Tube type (LMA vs ETT) had a negative regression coefficient, indicating a reduction in OR time (Tables 9, 10).

## Discussion

The investigation into the emergence times following general anesthesia, which compares the use of ETTs and LMAs, sheds light on a critical aspect of post-surgical care. The anatomical differences in airway management between these methods, with ETTs passing through the glottis into the trachea and LMAs resting above the glottis, prompted a comprehensive exploration of their respective implications on patient outcomes [9].

The literature review uncovered a nuanced landscape with studies presenting conflicting results on emergence times. While some studies suggested no significant difference between ETT and LMA emergence times, others hinted at potential advantages of LMAs, particularly in specific surgical scenarios. The need for a more robust comparison led to the initiation of this retrospective study, which uniquely taps into a high volume of surgeries across specialties at OhioHealth Doctors Hospital, providing a more comprehensive understanding of the dynamics between ETT and LMA usage.

The aims of the study were meticulously designed to dissect the relationships between airway management methods and various operative outcomes. The analysis of emergence times revealed a significant advantage for LMAs over ETTs, with LMAs associated with a 1.6-minute shorter emergence time. A possible explanation for this finding aligns with the hypothesis that the anatomical positioning of LMAs may facilitate faster removal post-surgery, although this hypothesis requires further investigation due to possible confounding factors. While the clinical significance of this shorter emergence time remains unclear, shorter emergence times have been associated with better post-operative neurological outcomes [10]. The incorporation of regression methods allowed for a deeper exploration, linking factors such as OR time, narcotics, and urological procedures as contributors to emergence time variations.

The comparison of broader operative data, including anesthesia duration, procedure time, and overall OR time, provided further insights. The results, particularly the negative regression coefficient for tube type (LMA vs. ETT) in the reduced model, indicate an association between LMA use and shorter emergence times, although this may be due to confounding factors rather than a direct effect of the airway device itself. Given the retrospective design, as well as the low R² value of the model, no causal relationship can be concluded. Furthermore, this finding may have implications for optimizing resource utilization and enhancing procedural efficiency, even though the study did not directly measure outcomes such as complications or case load. The association between LMA use and reduced overall operating times raises the possibility that the choice in airway device may contribute to improved hospital workflow efficiency [11,12]. Further research is needed to determine whether these associations are connected with clinically meaningful benefits. Examining the relationship between the use of ETTs and LMAs in the context of increased case load may provide an insight into improving provider efficacy, potentially alleviating the strain hospitals are facing regarding the increasing physician shortage.

The study also explored the association between emergence times and operative outcomes, hypothesizing that faster emergence might correlate with reduced anesthesia duration, procedure times, and overall OR times. Efficient management of anesthesia is a crucial aspect in how OR schedules are created [13]. Recent advancements in the field have been made with the goal of improving scheduling and reducing overall OR times [14]. The analysis supported this hypothesis, with variables such as regional anesthesia, narcotics, and emergence time positively contributing to OR time, while tube type (LMA vs ETT) exhibited a negative regression coefficient, indicating a potential reduction in OR time with LMA usage. However, due to the retrospective design and potential confounding factors, these findings should be interpreted with caution. Despite the study limitations, the results call attention to important associations and may serve as the foundation for future investigation on the topic. 

This study, while robust in its methodology and sample size, is not without limitations. The retrospective nature of the study introduces inherent biases and unmeasured confounders such as specific provider practices. Furthermore, the generalizability of findings beyond the specified patient population and time frame may be constrained. Additionally, the study does not delve into patient outcomes beyond operative and emergence times, leaving avenues for further research in areas such as postoperative complications and patient satisfaction, and personalized care [15].

## Conclusions

In conclusion, this study provides valuable insights into the potential advantages associated with LMA usage in terms of faster emergence times and its potential links to OR efficiency. Clinically, these results offer preliminary information to inform decision-making in airway management, with potential implications for optimizing surgical workflow and patient recovery. These findings contribute to the ongoing discourse on refining clinical practices in anesthetic management, emphasizing the need for tailored approaches in airway management based on surgical contexts and patient characteristics. Nevertheless, the multifaceted nature of perioperative care warrants ongoing investigation to unravel the intricate web of factors influencing these outcomes.
